# Outcomes of endovascular treatment of patients with intermittent claudication due to femoropopliteal disease

**DOI:** 10.1177/17085381211039668

**Published:** 2021-08-16

**Authors:** Ahmed A Naiem, Robert James Doonan, Oren K Steinmetz, Kent S MacKenzie, Elie Girsowicz, Jason P Bayne, Daniel I Obrand, Heather L Gill

**Affiliations:** 1 Division of Vascular Surgery, 5620 McGill University Health Centre, Montreal, QC, Canada; 2 Division of Vascular Surgery, Jewish General Hospital, Montreal, QC, Canada

**Keywords:** claudication, endovascular, revascularization, outcomes

## Abstract

**Objective:**

Our objective was to evaluate the outcomes of endovascular treatment in patients with moderate and severe claudication due to femoropopliteal disease, that is, disease of the superficial femoral and popliteal arteries.

**Methods:**

A retrospective review of all patients with moderate and severe claudication (Rutherford 2 and 3) undergoing endovascular treatment for FP disease between January 2012 and December 2017 at two university-affiliated hospitals was performed. All procedures were performed by vascular surgeons. Primary outcomes were mortality, freedom from reintervention, major adverse limb events defined as major amputations, open surgical revascularization, or progression to chronic limb-threatening ischemia (CLTI) at 30 days, 1 year, 2 years, and last follow-up. Unadjusted odds ratios were calculated to identify variables associated with adverse outcomes, and Kaplan–Meier survival curves were used to determine mortality and freedom from reintervention.

**Results:**

Eighty-five limbs in 74 patients were identified on review. Mean age was 69.6 ± 9.8 years and 74.3% were males. At a median follow-up of 49.0 ± 25.5 months, all-cause mortality rate was 8.1% (6 patients) with 16.7% being due to cardiovascular causes. Reintervention rates were 1.2%, 16.5%, and 21.2% at 30 days, 1 year, and 2 years, respectively. Major adverse limb events occurred in 3 patients and rates were 0%, 1.2%, and 2.4% at 30 days, 1 year, and 2 years, respectively. Progression to CLTI was 0%, 1.2%, and 1.2% at 30 days, 1 year, and 2 years, respectively. Claudication had improved or resolved in 55.6% (*n* = 34 patients), stable in 38.9% (*n* = 21 patients), and worse in 5.6% (*n* = 3 patients) Age ≥ 70 years (OR = 4.09 (1.14–14.66), *p* = 0.027), TASCII A lesion (OR = 4.67 (1.14–19.17), *p* = 0.025), and presence of 3-vessel runoff (OR = 3.70 (1.18–11.59), *p* = 0.022) predicted symptoms’ improvement. TASCII A lesions were less likely to require reintervention (OR = 0.23 (0.06–0.86), *p* = 0.020). Reintervention within 1 year (OR = 11.67 (0.98–138.94), *p* = 0.017), reintervention with a stent (OR = 14.40 (1.19–173.67), *p* = 0.008) and more than one reintervention (OR = 39.00 (2.89–526.28), *p* < 0.001) predicted major adverse limb events.

**Conclusions:**

Careful patient selection is important when planning endovascular treatment in patients with intermittent claudication and FP disease. This could result in symptomatic improvement in more than half of the patients. Adverse outcomes such as major adverse limb events, progression to CLTI, and amputations occur at low rates.

## Introduction

It is estimated that peripheral arterial disease (PAD) affects 200 million individuals worldwide.^1^ Symptomatic PAD most commonly manifests with intermittent claudication (IC) and has a relatively benign prognosis compared to the more severe chronic limb-threatening ischemia (CLTI).^2^ It is an important marker and predictor of adverse cardiovascular morbidity and mortality.^1,3–5^ Guidelines recommend optimal medical therapy (OMT) with antiplatelet agents, statins, and risk factors modification in all patients with PAD to reduce adverse cardiovascular events. Moreover, supervised exercise therapy (SET) is recommended as adjunct therapy for patients with IC.^2,6^ Serious challenges exist in both availability and accessibility of SET even in high-income countries.^7–10^ Revascularization through endovascular treatment (ET) in patients with IC can provide improvement for those with disabling symptoms.^6,11^ There are concerns about progression to CLTI and increased risk of limb loss as a result of the procedure.^12–15^ Our objective was to evaluate whether ET in patients with moderate and severe IC due to femoropopliteal (FP) disease results in increased adverse limb outcomes.

## Methods

### Study design

We conducted a retrospective review of all patients with moderate and severe IC resulting from FP disease undergoing ET between January 2012 and December 2017. This took place in two tertiary care university-affiliated health centers in Montreal, Quebec, Canada. Patients presenting with moderate or severe IC defined as Rutherford 2 and 3 grade, respectively, associated with FP disease on computerized tomography, magnetic resonance, angiography, or duplex ultrasonography were included. If a patient underwent ET in both legs, each leg was analyzed independently. Patients with CLTI, concomitant aorto-iliac, common femoral, and/or tibial vessel procedures were excluded. Necessary ethics approval was obtained through centralized research ethical board reviews. Demographic data and anatomical data were collected from each institute’s electronic health records. All angiograms were evaluated by the first author (senior vascular surgery resident) and an attending vascular surgeon. In addition, noninvasive vascular laboratory results were retrieved from our standard postoperative surveillance protocol records which is ankle-brachial index (ABI) and arterial duplex at 6 weeks postoperatively, ABI and arterial duplex every 4 months for 1 year, and then ABI and arterial duplex every 6 months thereafter. Time to intervention was defined as time from diagnosis of IC to ET. Primary outcomes were mortality, reintervention rates, major adverse limb event (MALE) (defined as major amputation or open surgical revascularization), Major adverse cardiovascular events (MACE; defined as nonfatal myocardial infarctions, nonfatal stroke, or cardiac-related deaths), minor amputations and progression to CLTI (defined as ischemic rest pain, or tissue loss). Secondary outcomes included adherence to OMT, improvement in symptoms defined as subjective reduction in pain with walking or increased walking endurance, factors associated with symptom improvement, factors associated with reintervention, and factors associated with MALE.

### Data analysis

Kaplan–Meier survival analyses were performed to determine the rate of reinterventions and mortality over time. The patterns of procedures for claudicants with FP disease (i.e., angioplasty, angioplasty with or without stent, and a drug-coated balloon) and its relation to postoperative outcomes were analyzed. Contingency tables and Pearson’s chi-square test were used for binary outcomes. Unadjusted odd ratios with 95% confidence intervals (CI) were calculated. Finally, Kaplan–Meier survival analyses were used to determine if the pattern of procedure leads to a difference in survival, reinterventions, MALE, and cardiovascular events over time. All analyses were performed in SPSS Version 27. *p* < 0.05 was used as marker of statistical significance.

## Results

**Figure 1.**
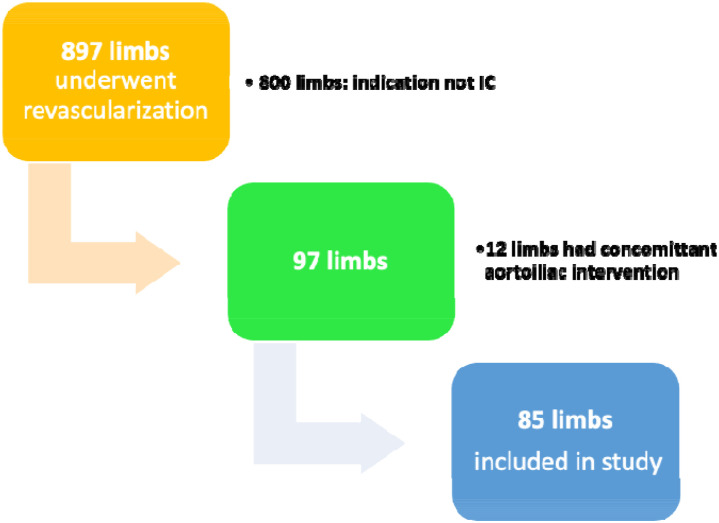
Results of retrospective review through 897 patients undergoing ER between January 2012–December 2017.

Eighty-five limbs in 74 patients were identified on review (Figure 1). Mean age of the population was 69.6 ± 9.8 years, and 74.3% were males. The most commonly encountered comorbidities were hypertension (74.3%), dyslipidemia (67.6%), and history of smoking (66.2%) (Table 1).

**Table 1.** Characteristics of patients undergoing ET for intermittent claudication.

Mean age in years, mean (SD)	69.6 (9.8), range = 53–91	
Sex, % (*n*)	Male	74.3% (*n* = 55/74)
	Female	25.7% (*n* = 19/74)
Comorbidities, % (*n*)	Hypertension	74.3% (*n* = 55/74)
	Diabetes mellitus	35.1% (*n* = 26/74)
	History of smoking	66.2% (*n* = 49/74)
	Dyslipidemia	67.6% (*n* = 50/74)
	Coronary artery disease	29.7% (*n* = 22/74)
	Stroke	16.2% (*n* = 12/74)
	Chronic kidney disease	1.2% (*n* = 1/74)
	Active or previous cancer	14.9% (*n* = 11/74)
	Severe comorbidities^a^	59.5% (*n* = 44/74)
Rutherford classification, % (*n*)	II	3.6% (*n* = 3/84)
	III	96.4% (*n* = 81/84)
Previous revascularization, % (*n*)	Ipsilateral	2.4% (*n* = 2/84)
	Contralateral	14.3% (*n* = 12/84)
Medications, % (*n*)	Antiplatelet	68.9% (*n* = 51/74)
	Statin	64.9% (*n* = 48/74)
	Anti-hypertensive	81.8% (*n* = 45/55)
	Anti-diabetic	65.4% (*n* = 17/26)
	OMT^b^	23.0% (*n* = 17/74)

^a^Defined as presence of three of the following: hypertension, diabetes mellitus, dyslipidemia, coronary artery disease, and stroke.

^b^OMT: optimal medical therapy defined as use of antiplatelets, statin, and non-smoking.

Lesions were characterized as TASC II A in 29.8%, B in 46.4%, and C in 23.8%. There were no TASCII D lesions treated. Forty-three patients (51.2%) were treated with a stent in their index procedure. The stents used were bare metal nitinol stents. Sizing was done via preoperative CT measurements, intraoperatively visual estimation or use of the hybrid room software. For patients who needed a stent as an adjunct to plain balloon angioplasty (PBA), the stent used was 1 mm larger than the balloon, that is, 6 mm stent post 5 mm PBA. One patient was treated with a drug-coated balloon (DCB). No patients received treatment with an atherectomy device. Intravascular ultrasound is not available in our practice (Table 2).

**Table 2.** Anatomical and ET procedure details.

TASC II Classification, % (*n*)	A	29.8% (*n* = 25/84)
	B	46.4% (*n* = 39/84)
	C	23.8% (*n* = 20/84)
	D	0%
Anatomy of lesions, % (*n*)	Location	
	Proximal SFA	19% (*n* = 16/84)
	Mid SFA	45.2% (*n* = 38/84)
	Distal SFA	70.2% (*n* = 59/84)
	Above-knee popliteal	16.7% (*n* = 14/84)
	Below-knee popliteal	2.4% (*n* = 2/84)
Infra-popliteal runoff, % (*n*)	3 vessels	48.2% (*n* = 40/83)
	2 vessels	37.3% (*n* = 31/83)
	1 vessel	14.1% (*n* = 12/83)
Primary procedure type, % (*n*)	Plain balloon angioplasty (PBA)	95.2% (*n* = 80/84)
	Drug-coated balloon (DCB)	1.2% (*n* = 1/84)
	Stent	3.6% (*n* = 3/84)
Angiogram findings, % (*n*)	Subintimal recanalization	37.8% (*n* = 31/82)
	Residual stenosis	51.9% (*n* = 42/81)
	Vessel dissection	38.8% (*n* = 31/80)
	Embolization	3.8% (*n* = 3/79)
Adjunct procedure required,% (*n*)	66.7% (*n* = 56/84)	
Adjunct procedure type, % (*n*)	PBA	21.4% (*n* = 12/56)
	Stent	76.8% (*n* = 43/56)
	Others^a^	1.8% (*n* = 1/56)
Mean stent length used in mm, mean (range)	162.8 ± 81.1 (37–350)	
Closure device success, % (*n*)	92.4% (*n* = 73/79)	

^a^Tibioperoneal trunk thrombolysis for embolization.

**Figure 2.**
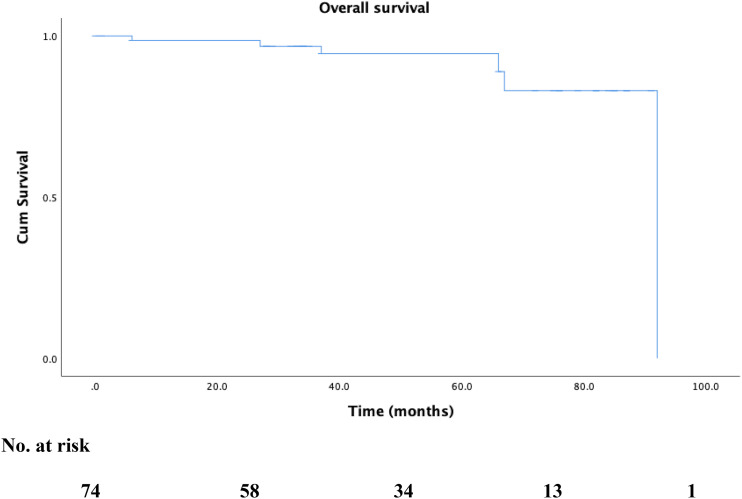
Kaplan–Meier survival curve for overall survival during follow-up.

**Figure 3.**
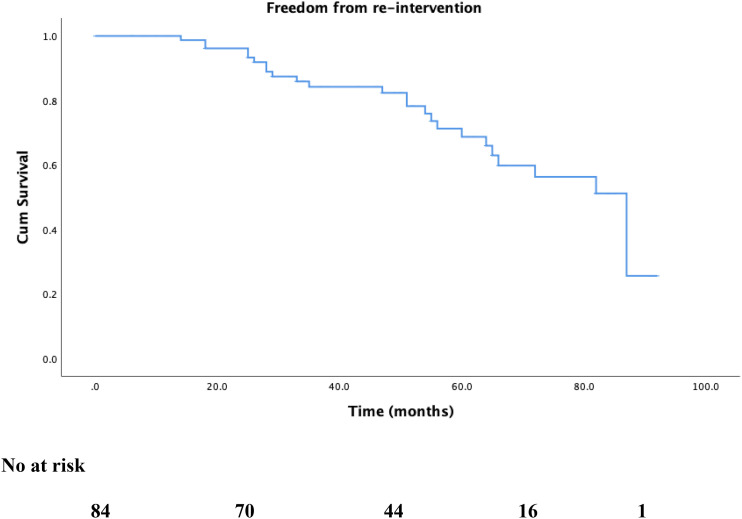
Kaplan–Meier freedom-from-reintervention curve during follow-up.

There were no mortalities recorded up to 2 years. At a median follow-up of 49.0 ± 25.5 months, all-cause mortality rate was 8.1% (6 patients). Figure 2 shows Kaplan–Meier survival estimates for overall survival. Cardiovascular causes accounted for 16.7%. Reintervention rates were 1.2%, 16.5%, and 21.2% at 30 days, 1 year, and 2 years, respectively (Figure 3). MALE occurred in 3 patients and rates were 0%, 1.2%, and 2.4% at 30 days, 1 year, and 2 years, respectively. The first patient’s index ET was an superficial femoral artery (SFA) angioplasty for a TASC II B lesion. Six months later, symptoms recurred with evidence of recurrent stenosis in the SFA and above-knee popliteal segments; this was stented. 2 years elapsed before the SFA stent thrombosed and the patient presented with CLTI grade 4 (rest pain). He underwent a femoral below-knee popliteal bypass. The second patient underwent SFA stenting which occluded 2 months later and presented with rest pain. He underwent a femoral above-knee bypass 10 months post index ET. The third patient had an initial SFA stent for a TASC II C lesion which occluded 15 months later and required angioplasty and stenting. This itself occluded 7 months later and ET was not possible. He underwent a femoral below-knee popliteal bypass 24 months after the index ET.

The rate of progression to CLTI was 0%, 1.2%, and 1.2% at 30 days, 1 year, and 2 years, respectively. Median ABI improvement was 0.11 and 0.29 at 1 year and 2 years, respectively. Claudication had improved or resolved in 55.6% (*n* = 54 patients), stable in (*n* = 21 patients), and worse in 5.6% (*n* = 3 patients) at a median follow-up of 49.0 +/− 25.5 months (Table 3).

**Table 3.** Outcomes in claudicants undergoing endovascular procedures for femoropopliteal disease.

Mortality rate. % (*n*)	30 days	0%
	1 year	0%
	2 years	0%
	Overall	8.1% (*n* = 6/74)
Cause of death. % (*n*)	Cardiovascular	16.7% (*n* = 1/6)
	Non-cardiovascular	66.7% (*n* = 4/6)
	Unknown	16.7% (*n* = 1/6)
Reintervention rates, % (*n*)	30 days	1.2% (*n* = 1/85)
	1 year	16.5% (*n* = 14/85)
	2 years	21.2% (*n* = 18/85)
	More than 2 years	29.4% (*n* = 25/85)
Median ABI, Mean ± SD (range)	Preintervention	0.64 ± 0.16 (0.27–1.00)
	At 1 month	0.95 ± 0.18 (0.48–1.35)
	At 2 months	0.96 ± 0.16 (0.48–1.14)
	At 6 months	0.84 ± 0.17 (0.51–1.00)
	At 12 months	0.75 ± 0.18 (0.42–1.00)
	At 18 months	0.80 ± 0.20 (0.40–1.02)
	At 24 months	0.93 ± 0.16 (0.50–0.99)
	At 60 months	0.85 ± 0.21 (0.68–1.21)
Major adverse limb events (MALE), % (*n*)	30 days	0%
	1 year	1.2% (*n* = 1/85)
	2 years	2.4% (*n* = 2/85)
	Overall	3.5% (*n* = 3/85)
Progression to CLTI, % (*n*)	30 days	0%
	1 year	1.2% (*n* = 1/85)
	2 years	1.2% (*n* = 1/85)
	Overall	3.5% (*n* = 3/85)
Minor amputations, % (*n*)	30 days	0%
	1 year	0%
	2 years	0%
	Overall	1.2% (*n* = 1/85)
Median follow up in months, mean ± SD (range)	49.0 ± 25.5 (0–92.0)	
Reported symptoms at last vascular follow-up % (*n*)	Improved or resolved symptoms	55.6% (*n* = 30/54)
	Stable	38.9% (*n* = 21/54)
	Worse	5.6% (*n* = 3/54)

Patients with symptomatic improvement had lower mean ABI prior to revascularization (0.58 vs 0.69, *p* = 0.014) and were more likely to have 3-vessel runoff (69% vs 38%, *p* = 0.022). Table 4 summarizes differences between the improved and stable/worse symptoms cohorts. Factors associated with improved symptoms were age ≥ 70 years (OR = 4.09 (1.14–14.66), *p* = 0.027), a TASCII A lesion (OR = 4.67 (1.14–19.17), *p* = 0.025), and presence of 3-vessel runoff (OR = 3.70 (1.18–11.59), *p* = 0.022). Medical comorbidities and adherence to OMT did not predict symptoms’ improvement (Table 5).

**Table 4.** Comparison of patients with improved symptoms versus stable or worse symptoms.

	Improved symptoms (*n* = 30)	Stable or worse symptoms (*n* = 24)	*p* value
Mean age in years, mean (SD)	70.0 (10.5)	66.7 (8.4)	0.243
Male sex, %	69%	75%	0.667
Mean preintervention ABI, mean (SD)	0.58 (0.14)	0.69 (0.15)	0.014
HTN, %	73%	80%	0.585
DM, %	42%	40%	0.875
Smoking, %	62%	65%	0.809
Dyslipidemia, %	65%	85%	0.133
CAD, %	27%	50%	0.108
Stroke, %	19%	10%	0.388
CKD, %	4%	0%	0.375
Active or previous cancer, %	8%	10%	0.783
Severe comorbidities, %^a^	54%	80%	0.065
Medications, %			
Antiplatelet agent	85%	75%	0.415
Statin	73%	70%	0.818
Previous intervention	0%	4%	0.259
TASC II classification, %	A 40%	A 13%	0.065
	B 40%	B 67%	
	C 20%	C 21%	
Presence of three vessel runoff, %	69%	38%	0.022
Reintervention within 1 year, %	13%	25%	0.273
Reintervention within 2 years, %	17%	38%	0.083
Mean ABI at 12 months, mean (SD)	0.74 (0.09)	0.84 (0.14)	0.343

^a^Defined as presence of three of the following: hypertension (HTN), diabetes mellitus (DM), dyslipidemia, coronary artery disease (CAD) and stroke SD: standard deviation; CKD: chronic kidney disease; TASC: Trans Atlantic Inter-Society Consensus.

**Table 5.** Unadjusted odds ratio for factors associated with improved symptoms in patients undergoing endovascular treatment.

Factor	Unadjusted OR (95% CI)	*p*-value
Age ≥ 70	4.09 (1.14–14.66)	0.027
Male sex	0.75 (0.20–2.78)	0.667
Severe comorbidities^a^	0.29 (0.08–1.11)	0.065
OMT^b^	1.80 (0.49–6.55)	0.373
TASCII A lesion	4.67 (1.14–19.17)	0.025
Subintimal recanalization	0.63 (0.20–1.95)	0.422
Residual stenosis	1.13 (0.38–3.38)	0.829
Vessel dissection	0.89 (0.30–2.66)	0.829
Use of stent in index ET	1.10 (0.38–3.26)	0.854
3-Vessel runoff	3.70 (1.18–11.59)	0.022
Reintervention	1.27 (752–2.15)	0.349
Time to intervention > 12 weeks	1.68 (1.08–2.62)	0.065

ET: endovascular treatment; CI: confidence intervals

^a^Defined as presence of three of the following: hypertension, diabetes mellitus, dyslipidemia, coronary artery disease, and stroke.

^b^OMT: optimal medical therapy defined as use of antiplatelets, statin, and non-smoking.

Patients undergoing ET for TASCII A lesions were less likely to require reintervention (OR = 0.23 (0.06–0.86), *p* = 0.020). Factors such as age, sex, comorbidities profile, and adherence to OMT did not predict reinterventions (Table 6). Moreover, adverse outcomes were more likely to occur in patients requiring reinterventions. Factors such as early reintervention within 1 year (OR = 11.67 (0.98–138.94), *p* = 0.017), reintervention with a stent (OR = 14.40 (1.19–173.67), *p* = 0.008), and more than one reintervention (OR = 39.00 (2.89–526.28), *p* < 0.001) predicted MALE (Table 7).

**Table 6.** Unadjusted odds ratio for factors associated with reintervention in patients undergoing endovascular treatment.

Factor	Unadjusted OR (95% CI)	*p*-value
Age ≥ 70	0.93 (0.33–2.59)	0.887
Male sex	0.74 (0.24–2.32)	0.604
Severe comorbidities^a^	2.59 (0.82–8.13)	0.098
OMT^b^	1.27 (0.36–4.48)	0.711
TASCII A lesion	0.23 (0.06–0.86)	0.020
3-Vessel runoff	0.62 (0.24–1.61)	0.327
Use of stent in index ET	1.11 (0.38–3.26)	0.854

ET: endovascular treatment; CI: confidence intervals

^a^Defined as presence of three of the following: hypertension, diabetes mellitus, dyslipidemia, coronary artery disease, and stroke.

^b^OMT: optimal medical therapy defined as use of antiplatelets, statin, and non-smoking.

**Table 7.** Unadjusted odds ratio for factors associated with major adverse limb events in patients undergoing endovascular treatment.

Factor	Unadjusted OR (95% CI)	*p*-value
TASCII A or B	7.00 (0.60–81.68)	0.076
No 3-vessel runoff	1.90 (0.17–21.83)	0.600
Vessel dissection	1.60 (0.10–26.55)	0.741
Adjunct intervention in index OR	1.00 (0.09–11.53)	1.000
Use of stent in index ET	1.95 (0.17–22.38)	0.585
Reintervention within 1 year	11.67 (0.98–138.94)	0.017
Reintervention with stent	14.40 (1.19–173.67)	0.008
Multiple reinterventions	39.00 (2.89–526.28)	< 0.001

ET: endovascular treatment; CI: confidence intervals.

## Discussion

This study reflects a contemporary Canadian experience with ET in patients with moderate-to-severe IC at two university centers. Our patients’ demographics including age, male predominance, and comorbidity profile were similar to already published randomized and non-randomized trials.^11,12^ Both the Society for Vascular Surgery and American College of Cardiology/American Heart Association (ACC/AHA) guidelines stress the importance of OMT with antiplatelets, statins, and smoking cessation.^2,6^ In our cohort, 68.9% of patients were on antiplatelets (aspirin, clopidogrel, or both) and 64.9% were on statins. This seemingly low adherence to OMT is similar to previous larger-scale studies that reported statin use as low as 11%.^12,13^ When analyzing use of antiplatelets, statins, and non-smoking status, only 23% were considered to be on OMT. SET is an important pillar in management of patients with IC with reported improvement in maximal walking distance, pain-free walking up to 15 months compared to OMT alone.^16–18^ In our practice, we advise home exercise therapy (HET) for all patients as there are no hospital-associated SET programs available within our province. While lacking the supervision involved in SET, HET has been shown to improve symptoms in a recent network meta-analysis involving 42 trials and 3515 patients.^19^ All patients are prescribed a single antiplatelet agent preoperatively and postoperatively. Moreover, patients who receive a stent of any kind are discharged on dual antiplatelet agents for 3 months, followed by a lifelong single antiplatelet agent.

Our FP anatomy and distribution of disease were comparable to previous studies with more TASC II A/B than TASC II C/D lesions.^16–18^ Previous studies show that long complex lesions involving below-knee popliteal segments fare worse and have lower patency.^12,14,20^ In our cohort, only 2.4% of the patients had below-knee popliteal artery involvement.

Intermittent claudication is an important marker of overall cardiovascular health^1,3–5^ with reported mortality of 10–15% in claudicants at 5 years.^2^ Our cohort had no procedure-related mortality, and 8.1% (6 patients) mortality at a median of 49.0 ± 25.5 months (0–92.0). Cardiovascular-related death was identified in one patient where death was due to congestive cardiac failure (16.7%) 6 years after the intervention. Two patients (33.3%) died secondary to lung cancer at 3 years post intervention, one patient with sepsis related to pneumonia 4 years post intervention (16.7%), and one patient with massive hemoptysis (16.7%) 7 years later. A cause could not be determined in the sixth patient who died 3 years post intervention. Overall, 66.7% of the deaths were non-cardiovascular in comparison to 33–57% reported in the literature.^12,13^

Despite numerous studies examining ET in IC, the balance between improvement in quality of life on one hand and procedure-related complications on the other is unclear.^11–16,19^ Our cohort had a reintervention rate of 1.2%, 16.5%, and 21.2% at 30 days, 1 year, and 2 years follow-up, which is lower than the 26–49% reintervention rate reported in the literature.^12,15,20^ A number of factors could contribute to the high reintervention rate in the reported literature including a tendency toward less stent use when the initial indication for revascularization was IC although stenting in FP disease improves primary patency.^21^ In our cohort, 46 limbs (54.8%) received stenting in their index procedure. It was primary, that is, intention to stent beforehand, in 6.5% (3 limbs) in comparison to the remainder 93.5% (43 limbs) who had a stent after balloon angioplasty was deemed suboptimal. Factors such as complex FP anatomy (17.9% in our cohort), more extensive subintimal recanalization (37.8%), and post-angioplasty dissection (38.8%) were indications for stenting but the association between intra-op findings and patency could not be ascertained by statistical interrogation in our cohort. These same factors reportedly contribute to reduced patency and increased adverse outcomes and reinterventions.^17–22^ A DCB was used in one patient with a distal SFA occlusion and severe IC in our cohort. In our institution, DCB and drug-eluting stents are usually reserved for CLTI for economic reasons. In our cohort, adverse outcomes occurred in 3 patients and were comparable to larger-scale reports. MALE rates were 0%, 1.2%, and 2.4% at 30 days, 1 year, and 2 years, respectively. This is similar to the 1.6–4% incidence reported with ET.^12,23^

Progression to CLTI was seen in 3.5% of the limbs undergoing ET, which is comparable to the reported natural history of patients with IC without intervention.^2^ A 2017 meta-analysis of randomized trials by Pandey et al.^11^ did not show an increase in amputations with ET in comparison to OMT and SET. A recently published experience with revascularization for IC by both open surgery and ET showed that patients’ comorbidities determine adverse outcomes such as CLTI and MALE. In our cohort that was treated exclusively by ET, we found that anatomical factors rather than patients’ baseline characteristics determined worse outcomes. Symptoms improvement was more likely in patients ≥ 70 years, TASC II A lesions, and limbs with 3-vessel run-off. Reintervention was less likely with TASC II A lesions, and MALE was more likely in patients with early and multiple reinterventions.

## Limitations

This is a retrospective, non-randomized study with a small cohort of 85 limbs. It represents a real-world description of outcomes in a system where SET is not available. There is no validated quality of life outcomes reported in our study.

## Conclusion

Careful patient selection is important when planning ET in patients with IC and FP disease. This could result in symptomatic improvement in more than half of the patients. Adverse outcomes such as MALE, progression to CLTI, and amputations occur at low rates.
